# A review of reviews exploring gender differences in sickness absence

**DOI:** 10.5271/sjweh.4321

**Published:** 2026-11-01

**Authors:** Amber R McKenzie, Bethany Croak, Lise Gulli Brokjøb, Sharon A M Stevelink, Ira Madan, Beate Brinchmann, Wendy Nilsen, Karen Walker-Bone, Nils Abel Aars, Arnstein Mykletun

**Affiliations:** 1 Centre for Health and Work, Nordland Hospital, Bodø, Norway.; 2 King’s Centre for Military Health Research (KCMHR), King’s College London, London, UK.; 3 Department of Psychological Medicine, King’s College London, London, UK.; 4 Centre for Population Health, Haukeland University Hospital, Bergen, Norway.; 5 Occupational Health Department, Guy’s and St Thomas’ NHS Foundation Trust Work.; 6 The Work Research Institute, Centre for Welfare and Labour Research OsloMet – Oslo Metropolitan University, Oslo, Norway.; 7 School of Public Health and Preventive Medicine, Monash University, Melbourne, VIC, Australia.; 8 Norwegian Institute of Public Health, Oslo, Norway.; 9 Department of Community Medicine, University of Tromsø, Norway.

**Keywords:** absenteeism, meta analysis, meta review, sick leave, systematic review, woman, workplace

## Abstract

**Objectives:**

Long-term sickness absence is a risk factor for leaving the workforce, causing economic stress and deteriorating health. Women have higher rates of sickness absence compared to men in most, but not all, European countries. Reasons for apparent gender differences are currently poorly understood, but hypotheses include that women may have more health problems (particularly those associated with reproductive health). Other hypotheses for gender differences include gendered health beliefs, attitudes/norms to sickness absence, work factors, work–family conflict, or barriers to return-to-work. This umbrella review aimed to synthesize and critically appraise the empirical evidence underpinning these hypotheses.

**Methods:**

Following PRISMA and Joanna Briggs Institute methodological guidelines, a systematic search of seven electronic databases was conducted for peer-reviewed reviews (eg, systematic reviews, scoping reviews, critical reviews) and meta-analyses reporting on gender and sickness absence. The review protocol was pre-registered on PROSPERO.

**Results:**

In total, 133 reviews were eligible for inclusion, of which 12 directly focused on gender differences in sickness absence. Only three hypotheses were investigated frequently and, although there was strong face validity, there was limited and mixed-quality underpinning empirical evidence. There was a surprising lack of focus on men and sickness absence.

**Conclusion:**

Despite much research, the underlying causes for a gender gap in sickness absence remain poorly understood. Our findings highlight the need for caution when interpreting proposed hypotheses as established explanatory evidence. Whatever the cause, any gender difference in sickness absence is a gender equality problem, regardless of the cause. Future research should aim beyond describing gender differences to critically evaluating risk factors to develop prevention strategies.

Average sickness absence days increased in Europe 2006–2020 (1), causing a costly and growing international burden in lost productivity (2). Comparing sickness absence internationally is challenging but taking sick leave is generally perceived to be in the public’s best interest (especially workers with infectious diseases) and serves as a safety net to ensure financial stability for ill employees while recovering (3), given that working whilst ill without necessary work adjustments can exacerbate symptoms (4). However, excessive sickness absence causes adverse impact for the individual (unwanted employment changes; social isolation; increased risk of permanent labor market exit) (5), the employer (disrupted organizational function, co-worker’s workload; productivity loss) and wider society (direct benefit costs and loss of tax income) (1, 6).

For these reasons, understanding risk factors for excessive sick leave is important to develop prevention strategies. However, sickness absence is complex. For example, when examining sickness absence attributed to musculoskeletal pain, more than 30-fold differences in the 12-month prevalence of sickness absence were found among 12 417 workers across 47 occupational groups from 18 countries (7). Even among office workers doing similar tasks, there was a 10-fold variation. Sickness absence rates differ by occupation (8), educational attainment, and ethnicity (9). Another risk factor is gender (7, 10), such that women generally have higher rates of sickness absence than men, with a gap that has been widening over time. Data from Europe 2006–2020 shows an average yearly decrease in costs for absenteeism among male workers (-0.3% each year), but an annual 0.5% increase among female workers (1).

Several theoretical frameworks have been proposed to understand sickness absence and generate hypotheses to understand the gender gap. Researchers argue that individual characteristics, societal influences and workplace factors impact sickness absence differently by gender (10–14). For instance, researchers have increasingly challenged the traditional separation of work and home domains, highlighting the complex interplay between occupational demands and personal life (15). An example of this is the Work–family Conflict Model, grounded in Role-Set Theory and Role Strain Theory (16, 17), which highlights that individuals navigate multiple, often conflicting, roles in the work and family domain, leading to strain. The Role Identity Model further expands on this, suggesting that identification with work and family roles influences one’s experience of conflict or balance, providing a hierarchy of priorities (18). Others propose that sickness absence can be primarily understood through a health perspective (19). A recent global study reported that generally, women tend to live longer than men but face higher levels of illness throughout life, including back pain, depressive disorder and headaches (20). Similarly, others argue that sickness absence could be attributed to women’s health (gynecological and reproductive health) (19). The reasons for gender differences have been the subject of conside rable debate, with researchers formulating a range of hypotheses. Therefore, given the growing focus on gender differences in sickness absence (21, 22) and the rapid growth of a complex body of literature, this paper aimed to conduct an umbrella review to synthesize the evidence underpinning existing hypotheses about gender differences in sickness absence from reviews and meta-analyses. The purpose of this umbrella review was to provide a clear overview of hypotheses rooted in existing literature and to identify gaps in knowledge to address in future research.

## Methods

This umbrella review followed PRISMA guidance for conducting systematic reviews (23) and the Joanna Briggs Institute (JBI) methodology for umbrella reviews (24). The protocol was registered with the International Prospective Register of Systematic Reviews (PROSPERO number: CRD42024570174)). One amendment to the original protocol was made regarding how the data was synthesized (see below).

### Search strategy

Systematic searches were conducted in July 2024 and updated in October 2025 across seven electronic databases (Embase, MEDLINE, Global Health, PsycINFO, Scopus, Web of Science, and ProQuest). Supported by a librarian, the research team collaboratively developed a search string. Reference lists of included reviews were also searched. Articles identified from the search were screened against predetermined eligibility criteria (supplementary material, www.sjweh.fi/article/4321, appendix A).

### Inclusion criteria

Reviews and meta-analyses were eligible for inclusion if articles reported on the exposure ‘gender’, the outcome ‘sickness absence’ and the eligible study design ‘review’, but the primary focus did not have to be sickness absence or gender differences. All peer-reviewed reviews (systematic, narrative, rapid, scoping) and meta-analyses were eligible, provided a systematic search for published articles in electronic databases was used. Umbrella reviews were excluded to reduce over-interpretation of the findings from the original studies. Reviews conducted in English, Dutch, Norwegian or Swedish were eligible. No time-period or country limits were imposed. Grey literature was excluded. Authors were contacted if full-texts could not be retrieved, but reviews were excluded if full-text was not provided.

For the umbrella review topic, it is critical to acknowledge the distinction between sex and gender. Sex can be defined as the biological categorization of males and females based on their reproductive mechanisms (25). Whereas gender is argued to be a social identification construct of either being a man or a woman, portraying masculinity or femininity, irrespective of reproductive organs. The umbrella review aimed to cover hypotheses spanning research that used ‘sex’ definitions for study samples (eg, health hypothesis), but also articles which focused on the social constructs of sickness absence and implemented ‘gender’ definitions (eg, attitudes and norms). Therefore, both definitions were included in this review.

### Screening

After searching, reviews were imported to EndNote and Covidence for eligibility screening; duplicates were removed. Given the breadth of the topic and the anticipated volume of reviews yielded, OpenAI (ChatGPT) was used to assist in the screening and basic data extraction processes following published guidance (26) (see supplementary appendix B for full methodology). AI was used to support the human reviewers, not replace judgment. Information relating to hypotheses about sickness absence gender differences was extracted manually by four reviewers from the research team.

### Quality assessment

After the full-text screening phase, all eligible reviews underwent quality assessment. The Critical Appraisal Skills Programme (CASP) Systematic Review Checklist (27) was used to assess the quality of each review. The quality assessment was carried out by human reviewers and supported by OpenAI. The human reviewer provided the CASP questions and the full article to the AI platform, and the human reviewer then reviewed and cross-referenced the completed quality assessment. A second reviewer independently assessed the quality of 10% of the articles to assess interrater reliability. Reviews were not excluded based on the quality; instead, the quality of existing reviews is reported.

### Data synthesis

A narrative synthesis was planned, given the heterogeneity of the aims and methodologies used in each review. The original aim was to extract researchers’ hypotheses from existing reviews and meta-analyses to critically appraise the supporting evidence, but many retrieved reviews did not include a hypothesis. To account for this, the published protocol was modified so when an a-priori hypothesis was stated, a deductive framework was used to guide analysis. When no hypothesis was stated, an inductive process was used to identify emerging hypotheses.

Initially, the research team developed a framework (table 1), including six sickness absence paradigms: (i) work–family conflict, (ii) gendered health, (iii) work factors, (iv) attitudes and norms, (v) health beliefs, and (vi) barriers to return to work (eg, return to work interventions).

**Table 1:** Framework for deductive analysis of reviews or meta-analyses with stated a priori hypotheses

Hypothesis	Description	Hypothesis supported?
Work–family conflict	Women often face greater demands in balancing work and family responsibilities, which may increase stress and lead to higher rates of sickness absence (15)	Review findings were mixed (N=8). Some articles found increased rates of work–family conflict for women, but men also reported experiencing work–family conflict.
Health	Biological factors such as reproductive health issues and other gender-specific vulnerabilities may lead to more frequent or prolonged sickness absence in women (20, 166).	Findings were mixed (N=94), with both genders taking more sickness absence comparatively for health conditions compared to ‘healthy’ comparison groups. There was more of a focus on women’s reproductive health on sickness absence than men’s reproductive health.
Work factors	Women may experience poorer working conditions, including bullying, harassment, fewer job opportunities, and higher emotional labour, contributing to sickness absence (160–162).	Both men and women were reported to experience increased sickness absence due to work factors (N=45), specifically adversities faced at work such as bullying or harassment.
Attitudes and norms	Gendered expectations and social norms (e.g., women being more accepting of taking leave) may influence sickness absence (22, 156, 163).	Most reviews provided support for gendered attitudes and norms (N=7). Expectations perceived by men and the role of the ‘male provider’ influenced reduced rates of sickness absence for men.
Health beliefs	Differences in perceptions about the benefits of sickness absence such as seeing sickness absence as necessary for recovery, may vary by gender (164).	Limited reviews investigating health beliefs (N=2). However, support that women tend to believe sickness absence was good for health and will allow workers recover and return back to work faster.
Barriers to return to work	Women may face more obstacles when trying to return to work, including physical limitations, psychological factors, workplace issues, and lack of support. This also encapsulates return-to-work interventions with the hypothesis that these interventions are not equally effective across genders (165).	There was moderate support from reviews (N=16) with reviews reporting that men return back to work faster following an illness. Further, women utilise time on sickness absence to balance domestic responsibilities so are less motivated to return faster.

Each included review was categorized by one or more hypotheses either if they explicitly named the hypothesis a-priori or investigated an associated risk factor. For example, a review of gender differences in sickness absence from cancer was categorized under ‘health’. Additional hypotheses identified but not specified a-priori were also extracted. There was an opportunity for the list of hypotheses to increase when new hypotheses were identified.

Finally, included reviews were used to explore whether there were any chronological trends of research topics reviewed and to identify any limitations (eg, quality issues) or gaps in the literature to create a road map for future research.

## Results

In total, N=2721 articles were retrieved from searches. After de-duplication (N=397), 2324 eligible reviews remained. After full-text screening, 661 reviews were assessed for inclusion (figure 1). Of these, 133 eligible reviews and meta-analyses, published 2000-2024, were included and are shown in table 2. Reviews included studies conducted mostly in Europe (95%), the United States and Canada. Almost all were systematic reviews (N=103), and 22 conducted meta-analyses. Three were described as critical reviews, one review was a meta-ethnography of qualitative studies, and another self-identified as a meta-synthesis of qualitative studies. Nearly half (N=66) did not use, or report a formal quality assessment tool. Not all reviewers described the study types eligible for inclusion, but among those which did, 94 included quantitative studies only, four included qualitative studies only, and 31 included quantitative, qualitative and mixed-methods studies.

**Figure 1:**
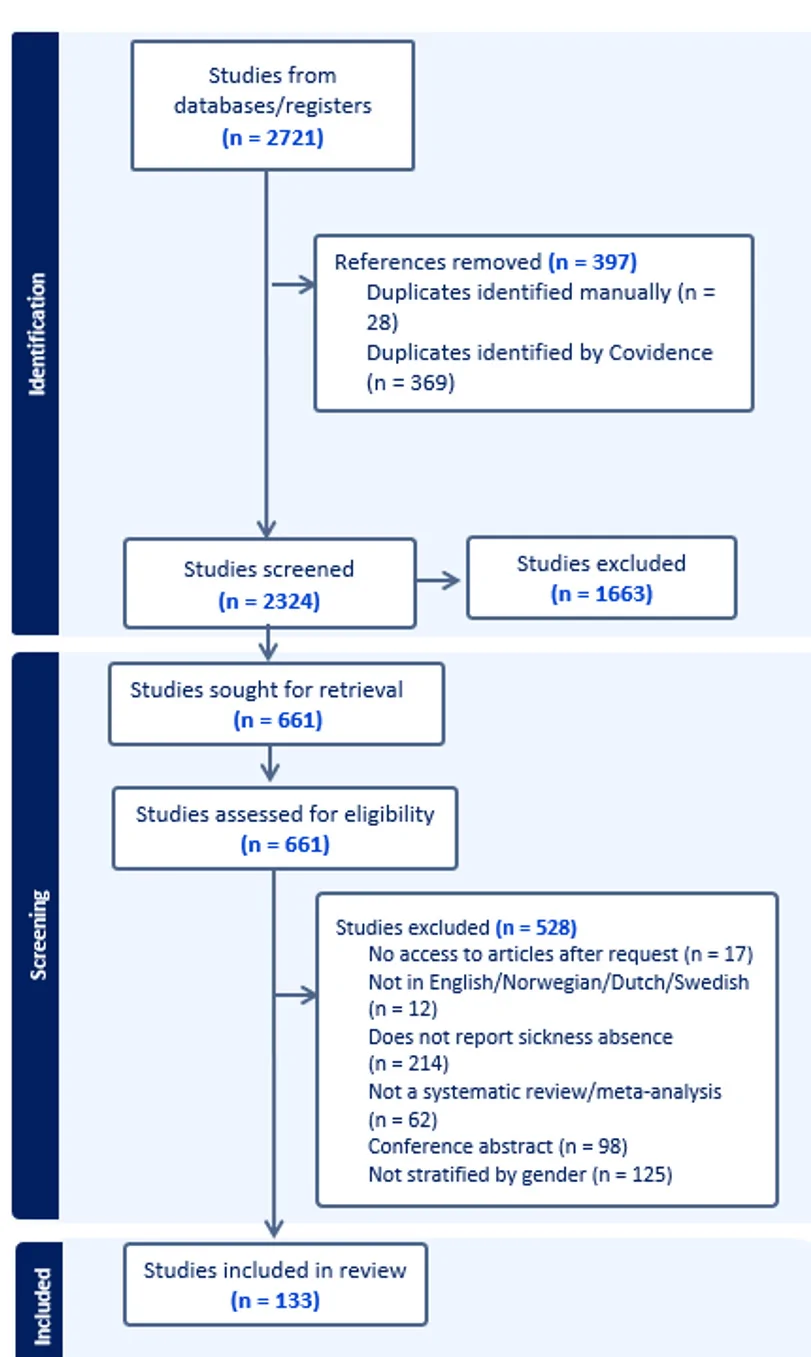
The PRISMA flow diagram demonstrating the process of study selection for the umbrella review of included reviews and meta-analyses (N=133).

**Table 2:** Full list of final included reviews and meta-analyses in the umbrella review (n = 133). [MA=meta-analysis; SLR=scoping literature review; SR=systematic review; MM=mixed methods; NR=not reported; QUAL=qualitative; QUAN=quantitative; RTW; return to work; WE=work environment.]

Author (publication date)	Title	Review type	Population	Studies (N)	Type of studies included	Hypothesis category	Key findings related to categories
Akay & Ahmadi (2022)	The work environment of immigrant employees in Sweden - a systematic review	SR	Immigrant workers based in Sweden	16	QUAN, QUAL & MM	WE	Female foreign-born workers with high burnout scores were associated with frequent sick leave. The authors noted this was not impacted by their immigrant status.
Almari et al (2021)	Quality of Life (QoL) among health care workers with diabetes mellitus: A literature review	SLR	Healthcare workers with diabetes mellitus	27	QUAN, QUAL & MM	Health	One study found that both men and women showed substantially higher hazard ratios for sick leave suffering from type 1 DM when compared to type 2.
Alyousef et al (2024)	The effect of lower limb osteoarthritis on work-related outcomes: a systematic review and meta-analysis	SR & MA	Working-age population (18–75 years) with osteoarthritis (OA)	7	QUAN	Health	Both male and female workers with OA undergoing surgery took more sick leave days per month when compared to controls.
Amiri & Behnezhad (2019)	Body mass index and risk of sick leave: A systematic review and meta-analysis	SR & MA	Working-age population	23	QUAN	Health	Both men and women were shown to be at greater risk of sick leave due to being obese or overweight. Men and women were not compared in the analysis, but women had a marginally increased RR.
Amiri & Behnezhad (2021)	Depression symptoms and risk of sick leave: a systematic review and meta-analysis	SR & MA	Working-age population with and without depression	15	QUAN	Health	Both men and women with depression experience more sick leave when compared to the general population. However, the findings suggest that men with depression are at a higher risk of sick leave than women.
Amiri & Behnezhad (2020)	Association between job strain and sick leave: a systematic review and meta-analysis of prospective cohort studies	SR & MA	Working-age population	18	QUAN	WE	Both men and women were shown to be at greater risk of sick leave due to job strain compared to controls; however, this was greater for men.
Amiri & Behnezhad (2020)	Sleep disturbances and risk of sick leave: systematic review and meta-analysis	SR & MA	Working-age population	11	QUAN	Health	Sleep disturbances increase the risk of sick leave for both genders compared to controls. No significant difference was found between men and women.
Amiri & Behnezhad (2020)	Alcohol consumption and sick leave: a meta-analysis	SR & MA	Working-age population	21	QUAN	Health	For both men and women, alcohol use increases the risk of sick leave by up to 11% and 10%, respectively.
Andersen et al (2023)	Barriers and facilitators to return to work following cardiovascular disease: a systematic review and meta-synthesis of qualitative research	SR & MA	Working-age population with cardiovascular disease	15	QUAL	Attitudes & norms	Male stereotypical gender roles (i.e., the breadwinner or the pillar of the family) were identified as encouraging faster return to work and reduced sick leave. Men may feel more obliged to return to work.
Armstrong et al (2022)	Economic burden of chronic hand eczema: A review	SR	Patients with chronic hand eczema	30	QUAN	Health	Women (24.5%) with chronic hand eczema had more prolonged sick leave than men (10.7%).
Bambra et al (2007)	The psychosocial and health effects of workplace reorganisation. 2. A systematic review of task restructuring interventions	SR	Working-age population	19	QUAN & MM	Health WE RTW barriers	Work task and role restructuring interventions led to reduced depression and sick leave for men.
Banning (2011)	Employment and breast cancer: A meta-ethnography	Meta- ethno-graphy	Working-age population with breast cancer	10	QUAL studies	Health Health beliefs WE RTW barriers	Returning to work for breast cancer patients was an important moment as it meant returning to normality and a distraction from life with cancer. Being back at work following sick leave provided a sense of structure, belonging, identity and social connectedness Financial issues increased pressure to return to work, with some women missing treatment cycles to be back in work. Fears around sick pay meant women would prefer to make work adjustments for treatment cycles rather than be on sick leave. Some women found their workplace to be supportive after long-term sickness absence, but this varied, as other women recovering from breast cancer perceived hostility from their employer following sickness absence.
Barkun & Leontiadis (2010)	Systematic review of the symptom burden, quality of life impairment and costs associated with peptic ulcer disease	SR	Working-age population with peptic ulcer disease	30	QUAN	Health	Men had greater sickness absence than women due to ulcers. Explained by medication changes for ulcers during this time and the fact that men are more susceptible to ulcers.
Beard et al (2009)	Economic and quality of life outcomes of antiretroviral therapy for HIV/AIDS in developing countries: A systematic literature review	SR	Populations from developing countries	16	QUAN & QUAL	Health	Receiving the correct antiretroviral therapy for aids/HIV improves sick leave rates for women but not for men.
Bekker et al (2009)	Sickness absence: A gender- focused review	Narrative literature review	Working-age population	NR	QUAN & QUAL	Health Attitudes & norms Work– family conflict WE	Sex differences between men and women, such as pregnancy and menstruation, contribute to gender differences in sickness absence. Stereotypical gender role and role orientation effects. Sickness absence linked to expressivity, thought of as feminine in the original article. Work-related gender-role stress and sex differences in coping with stress result in the sickness absence gender gap. Women often take on more of the caregiver role, causing work–family conflict and leading to more sickness absence. Low levels of coworker support are related to higher sickness absence for men, but supervisor support predicts absenteeism in some service occupations. Women are more likely to take sick leave due to work stressors such as noise and job complexity. However, men and women both respond to physical demands the same. Impact if gendered occupations where women are a minority and often do worse, resulting in greater sick absence.
Bernstrom & Houkes (2018)	A systematic literature review of the relationship between work hours and sickness absence.	SR	Working-age population	70	QUAN	WE	They found that part-time employees are significantly more likely to be absent and have longer absences, though the latter is only significant for women. Mixed findings were reported for pregnant women, showing both positive and negative associations with working full-time and sickness absence. Similar mixed findings were reported for women in general (not just pregnant) One study controlled for chronic disease in pregnant women and found a negative association with long work hours and sickness absence.
Bijker et al (2018)	Functional impairments and work-related outcomes in breast cancer survivors: A systematic review	SR	Working population of breast cancer survivors	20	QUAN & QUAL	Health WE	Functionality status was associated with less sickness absence and higher productivity for women with breast cancer. Shoulder function impacted prolonged sick leave. Better social functioning improved return-to-work rates. In general, impaired functioning was the driving factor for work or taking sick leave immediately following diagnosis, during treatment and in recovery.
Biswas et al (2022)	Differences between men and women in their risk of work injury and disability: A systematic review	SR	Working-age population	33	QUAN	WE	Noise was associated with long-term sickness absence for women only. Mixed findings for physical demands, where both men and women were associated with sickness absence. Physical violence/sexual assault and physical demands were associated with sickness absence, but no difference between men and women. No difference between men and women in sickness absence following repetitive work tasks, but both genders were associated. Bullying, job strain, organisational support and work stress are associated with sickness absence, but no difference between men and women
Blindow et al (2024)	Gender-based violence and harassment at work and health and occupational outcomes. A systematic review of prospective studies	SR	Working-age population	29	QUAN	WE	Most studies do not show any gender difference between harassment/unwanted sexual attention and sickness absence. One study showed sickness absence had a stronger association for men than women. Sexual harassment at work has a similar risk ratio for both men and women. One study found that men were at an increased risk of sickness absence following unwanted sexual attention at work.
Boonen (2006)	A review of work-participation, cost-of-illness and cost-effectiveness studies in ankylosing spondylitis	Narrative review	Working population with ankylosing spondylitis	NR	QUAN & QUAL	Health	Ankylosing spondylitis was associated with sickness absence for women.
Bravo et al (2024)	Methodological considerations in the study of perceived discrimination at work and its association with workers health and occupational outcomes: A scoping review.	SLR	Working-age population	102	QUAN, QUAL & MM	WE	Women and minority groups were disproportionately affected. 67.7% of studies reported that sex/gender predicted workplace discrimination, with women more affected. Some findings showed that women were up to 12 times more likely than men to perceive discrimination, and female surgeons were three times more likely to report discrimination. Discrimination is suggested to be a mediator for increased sick leave among women. The authors did not directly assess gender differences in absence due to discrimination.
Brunner et al (2023)	A narrative review on factors associated with job interruption during pregnancy.	Narrative literature review	Pregnant working population	42	QUAN, QUAL, and 5 literature reviews	Health WE Work– family conflict Health beliefs Other (social vulnerability, education)	Pregnancy-related conditions (e.g., pelvic girdle pain, nausea, fatigue, and pre-existing musculoskeletal pain) were strongly linked to absence. Sickness absence rates of pregnant workers were reported as higher in female-dominated workplaces when compared to male-dominated workplaces. Poor health and depression were risk factors for work absence during pregnancy. Job instability, temporary contracts, strenuous work, and workplace discrimination increased the risk of sick leave. Absence was more likely among women with multiple children, low social support, younger age (in some countries), and in female-dominated workplaces. National-level sick leave policies, occupational demands, and healthcare provider roles also shaped sick leave outcomes. Higher education was associated with long-term absence, while permanent contracts reduced absence. Country-specific norms and physician decisions also influenced pregnancy-related sick leave.
Burström et al (2011)	How equitable is vocational rehabilitation in Sweden? A review of evidence on the implementation of a national policy framework.	SR	Swedish general working population	11	QUAN	RTW barriers WE	Women were less likely to return to work after vocational rehabilitation than men. Men benefited more from intensive rehabilitation despite similar conditions (e.g., musculoskeletal disorders). Women received job training more often, while men were more likely to receive an education. Women’s lower return to work was potentially influenced by lower job adjustment possibilities, more domestic duties, and occupational roles with less autonomy.
Cancela-Carral et al (2022)	Therapeutic aquatic exercise in pregnancy: A systematic review and meta-analysis.	SR & MA	Pregnant working population	17	QUAN	Health	Aquatic exercise (water-based aerobic activity) was significantly more effective than land-based exercise in reducing pregnancy-related lower back pain and sick leave.
Chaker et al (2015)	The global impact of non-communicable diseases on macro-economic productivity: A systematic review.	SR	General population with non-communicable diseases	126	QUAN	Health Other (race and cultural return-to-work differences)	One study reported that white women were more likely than black women to return to work after a breast cancer diagnosis (74.2% vs. 59.6%), and black women were more likely to report sick leave (40.4% vs. 25.8%).
Chakraborty et al (2024)	Exploring the labour market outcomes of the risk factors for non-communicable diseases: A systematic review.	SR	General population with non-communicable diseases	109	QUAN	Health	In Belgium and Germany, overweight and obesity were significantly associated with increased sick leave for women (up to 47% and 41%, respectively), while findings for men were less consistent or not significant. In Denmark, diabetes was associated with higher sick leave among men (64%) than women (46%).
Ching et al (2023)	Biopsychosocial, work-related, and environmental factors affecting work participation in people with osteoarthritis: A systematic review.	SR	Working population with osteoarthritis (OA)	19	QUAN	Health WE	Women had a higher incidence of OA, particularly in healthcare, childcare, and cleaning sectors, where the risk of sickness absence was 3x higher than in administrative roles. For men, sectors like farming, transport, and construction also showed elevated OA-related absenteeism, but at a lower magnitude than women in similar high-demand roles.
Chow et al (2014)	Development of a conceptual framework to understand factors associated with return to work among cancer survivors: A systematic review.	SR	Working population of cancer survivors	27	QUAN & QUAL	Health	Return to work rates were lower among older breast cancer survivors. Chemotherapy and impaired work ability were barriers, especially for women with breast or colorectal cancer. Men scored higher on work ability up to a year post-sick leave. Some associations between gender and return to work were noted for specific cancers (e.g. blood malignancy and genital cancers).
Clevis et al (2025)	Are health-related, lifestyle, work-related, and socio-demographic factors associated with work productivity among menopausal women? A systematic review	SR	Working population of menopausal women	29	QUAN	Health	Associations between menopausal symptoms and absenteeism. Other factors were also associated with absenteeism including vasomotor symptoms, sleep quality and social support.
Cohen et al (2024)	Prevalence and burden of migraine in the United States: A systematic review.	SR	General population with migraines	26	QUAN	Health	73.6% of women and 56.4% of men reported ≥1 missed workday due to migraine over the past year. However, in other studies (e.g., AMS II, Baltimore Diary Study), men reported slightly more missed days in shorter time frames.
Collie & Gray (2022)	The relationship between work disability and subsequent suicide or self-harm: A scoping review.	Scoping review	Working-age population with work disability	47	QUAN & QUAL	Health Other (gender differences in non-work-related outcomes of sickness absence)	Longer durations of sickness absence were associated with an increased risk of suicide and self-harm in both men and women. Mixed findings suggested the effect was stronger in men, while others showed high risks among women-particularly those with prior mental health conditions.
Cornelius et al (2011)	Prognostic factors of long-term disability due to mental disorders: A systematic review.	SR	General population with mental health conditions	7	QUAN	Health	No significant effects of gender were found on return-to-work or disability outcomes in four of the included studies. One study did not explore gender, and another found a non-significant gender effect on the course of depression.
Côté & Coutu (2010)	A critical review of gender issues in understanding prolonged disability related to musculoskeletal pain: How are they relevant to rehabilitation?	Critical literature review	Working population with prolonged disability due to musculoskeletal (MSK) pain	31	QUAL and quantitative	Health Work–family conflict	Gender roles influence how prolonged MSK pain is experienced and managed. Women with multiple social roles (e.g., caregiver, homemaker, employee) were more likely to delay return to work. Narratives from women indicated different psychosocial coping mechanisms (crisis, breakpoint, migration) in response to long-term sickness absence.
de Oliveira et al (2023)	The role of mental health on workplace productivity: A critical review of the literature.	Critical review	Working-age population	38	QUAN	Health	In studies focusing exclusively on women, including low-income mothers, reduced psychological health significantly predicted workplace absence.
de Vries et al (2018)	Determinants of sickness absence and return to work among employees with common mental disorders: A scoping review.	SLR	Working population with common mental disorders (CMDs)	71	QUAN	Health	Female gender was identified in at least three studies as a predictor of sickness absence (SA) for people with CMDs, but not consistently for return to work. Lack of supervisor support doubled the risk of absence for both genders. While women are more frequently affected by common mental disorders, gender differences in return to work or sickness absence recurrence were inconsistent.
Dewa et al (2014)	Incidence rates of sickness absence related to mental disorders: A systematic literature review.	SR	Working population with mental disorders	10	QUAN	Health	Women had consistently higher sickness absence incidence rates related to mental disorders such as depression and psychosis across included studies.
Doran et al (2019)	A review of the economic impact of mental illness.	Rapid literature review	The general population with mental illness	45	QUAN	Health	One study noted that clinical mental disorders were associated with an increased risk of psychiatric sickness absence in men but not among women after adjusting for covariates.
Dorrington et al (2018)	Systematic review of fit note use for workers in the UK.	SR	UK working-age population	7	QUAN	Attitudes & norms	Fit notes were more frequently issued to women, especially for mental and behavioural disorders. Women were also more likely to receive ‘maybe fit’ notes. Female GPs were less likely to issue long-term fit notes for depression to female patients.
Duijvenbode et al (2009)	The relationship between overweight and obesity, and sick leave: a systematic review.	SR	Working-age population who are obese and/or overweight	13	QUAN	Health	Obesity was linked to increased sickness absence for both men and women, but the effect was often greater in men. However, obese women were also shown to have higher frequencies and durations of both short- and long-term sickness absence in some studies. Risk ratios and odds ratios varied but consistently suggested elevated risks across both sexes.
Elser et al (2018)	Blue-collar work and women’s health: A systematic review of the evidence from 1990 to 2015.	SR	Blue-collar working population	177	QUAN	WE	Women in blue-collar roles had significantly higher rates of sickness absence compared to women in other occupational groups. Female blue-collar workers had elevated risks for short- and long-term SA due to infection, musculoskeletal disorders, trauma, poor workplace climate, and domestic burden.
Ervasti et al (2017)	Prognostic factors for return to work after depression-related work disability: A systematic review and meta-analysis.	SR & MA	Working population with depression-related work disability	15	QUAN	Health	Meta-analysis yielded a pooled relative risk (RR) of 1.02 (95% CI: 0.98–1.05) for women, suggesting no sex difference in depression related return to work.
Etuknwa et al (2019)	Sustainable return to work: A systematic review focusing on personal and social factors.	SR	Working population on sick leave	79	QUAN, QUAL & MM	Health	For musculoskeletal conditions and common mental disorders, the findings were mixed, with some studies showing higher rates in return to work for men, and other studies favoured women.
Feijó et al (2019)	Risk factors for workplace bullying: A systematic review.	SR	Working-age population	51	QUAN	WE	The review found that women were more likely to be bullied at work, with odds ratios ranging from 1.17 to 2.77 across studies. Eleven studies reported no gender association.
Figueredo et al (2020)	Well-being at work after return to work (RTW): A systematic review.	SR	Working population who have returned to work after sickness absence	20	QUAN & QUAL	RTW barriers	Women were found to report slightly better psychological well-being and job satisfaction following return to work compared to men. Some studies noted that women had slightly more work modifications.
Finch (2003)	Pregnancy during residency: A literature review.	Narrative literature review	Pregnant physicians	29	QUAN & QUAL	Health WE	Despite intense working conditions and expectations, many pregnant residents had minimal sickness absence before delivery. This may be due to internalised guilt, workplace culture, and fear of burdening colleagues.
Fisker et al (2022)	Predictors of return to work for people on sick leave with common mental disorders: a systematic review and meta-analysis.	SR & MA	Working population on sick leave with CMDs	32	QUAN	Health WE	Women had a higher overall probability of returning to work with CMDs compared to men: OR = 1.16 (95% CI: 1.01–1.32). However, subgroup analyses (e.g. depression, specific time points) showed no significant sex-related differences.
Furlan et al (2022)	Primary care physicians’ learning needs in returning ill or injured workers to work. A scoping review.	SLR	Practitioners and working population returning from illness/injury	44	QUAN, QUAL, MM	Health Other (GP treatment approaches)	GPs often prescribe sick leave as a treatment for pregnancy-related conditions such as nausea, particularly due to reluctance to medicate. Highly educated women were the largest group of sickness absentees, and less likely to receive medication. Sick leave was seen as a safe alternative.
Gane et al (2021)	The impact of musculoskeletal injuries sustained in road traffic crashes on work-related outcomes: A systematic review	SR	Working population with musculoskeletal (MSK) injuries following a car accident	53	QUAN	Health	Two studies reported that women were more likely to take time off work or sick leave after a car accident. Two studies also found women took longer sick leave than men. Gender was inconsistently associated with work ability, work capacity, or return to work.
Gjellestad et al (2023)	Women’s health and working life: A scoping review	SLR	Working women	130	QUAN, QUAL & MM	Health	Women’s hormonal changes, pregnancy, infertility, and chronic conditions affect work participation. PMS and menstruation linked to absenteeism; pregnancy-related sick leave is a major contributor to the gender gap in sickness absence. Education level and age influence sick leave rates. No studies addressed abortion at work. Limited understanding of short-term sickness absence.
Gohar et al (2021)	Demographic, lifestyle, and physical health predictors of sickness absenteeism in nursing: A meta-analysis	Meta-analysis	Nursing population	17	QUAN	WE Attitudes & norms	Female nursing staff had significantly higher odds of sickness absenteeism compared to males. Explanations included higher self-reporting of health concerns among female staff, work–family conflict, and stigma preventing male nurses from taking sick leave.
González-Siles et al (2022)	Sense of coherence and work stress or well-being in care professionals: A systematic review	SR	Healthcare professionals	41	QUAN	WE	Higher sense of coherence (SOC) and improved organisational work environments were associated with reduced sick leave in female nurses. A stronger SOC helps individuals better manage stress, contributing to fewer absences.
Greener & Guest (2005)	Do antidepressants reduce the burden imposed by depression on employers?	SR	Working population with depression	Not specified	NR	Health	Depression significantly contributes to absenteeism, poor morale, and reduced productivity. Women are about twice as likely as men to be diagnosed with depression.
Halicka et al (2022)	Systematic review and meta-snalysis of predictors of return to work after spinal surgery for chronic low back and leg pain	SR & MA	Working population who have undergone spinal surgery for chronic low back and/or leg pain	8	QUAN	Health	No significant sex differences in return to work outcomes. Odds ratios for gender were not statistically significant.
Hansson & Jensen (2004)	Sickness absence due to Back and neck disorders	SR	Working population with neck and back pain	48	QUAN	Health	Women have a higher prevalence of neck disorders (40% vs. 29% in Norway). A Dutch registry study indicated palpable trapezius muscle pain among women as a risk factor for sick leave.
Harris & Hoggon (2015)	Hip osteoarthritis and work	Narrative SR	Working population with hip osteoarthritis	30	QUAN	Health	Rates of hip OA are higher among women (0.98%) than in men (0.70%). Among those with hip OA, 18.6% had missed work, and 44% had needed periods of sick leave.
Henning et al (2017)	Workplace harassment among staff in higher education: A systematic review	SR	Working population in higher education	51	QUAN, QUAL & MM	WE	Workplace harassment was predominantly reported by women (90% in one study), and is associated with increased absenteeism, job dissatisfaction, and staff turnover.
Henrotin & Gulisano (2022)	Sick leave during pregnancy and occupational factors: A systematic review	SR	Pregnant working women	11	QUAN	Health WE Work– family conflict	High rates of sick leave (SL) during pregnancy are linked to health conditions, work stressors (e.g. job strain, low support, physical workload), and work–family conflict. Withdrawal from work is seen as a protective strategy when adjustments are not possible.
Hensing & Wahlström (2004)	Sickness absence and psychiatric disorders	SR	Working population with psychiatric disorders	28	QUAN	Health	Women had more frequent sick-leave spells and a higher incidence of sickness absence due to psychiatric disorders (e.g. neurosis) than men, although men had longer average sick-leave durations for some diagnoses. Results varied across studies and countries, with some showing no sex differences.
Hensing et al (2023)	Domestic factors as determinants of sickness absence with psychiatric disorders: A scoping review of Nordic research published between 2010–2019	Scoping review	Nordic working population with psychiatric disorders	12	QUAN	Health Work– family conflict	Women consistently outnumber men in sickness absence with psychiatric disorders. Women face higher exposure to domestic factors, and some studies showed associations between domestic factors and sickness absence, though not all found gender-specific effects.
Hills & Joyce (2013)	A review of research on the prevalence, antecedents, consequences and prevention of workplace aggression in clinical medical practice	Narrative literature review	Clinical practitioners	Not specified	QUAN & QUAL	WE	Workplace aggression is linked to sick leave and absenteeism, with findings mixed when comparing men and women. Men were more likely to experience verbal threats and physical assault, while women were more likely to experience bullying and sexual harassment. No direct gender-specific analysis of aggression’s impact on sick leave was provided.
Huysmans et al (2018)	Return to work following surgery for lumbar radiculopathy: A systematic review	SR	Working population post-surgery for lumbar radiculopathy	63	QUAN	Health WE	Male sex was a consistent predictor of earlier return to work.
in’t Hout et al (2024)	Factors related to the recurrence of sickness absence due to common mental health disorders: A systematic review	SR	Working population with common mental disorders (CMD)	20	QUAN & QUAL	Other (gender and recurrent sickness absence)	While previous research suggests women experience more sickness absence, this review did not find consistent gender differences in recurrent sickness absence (RSA). One study found that married women were more likely to experience RSA than unmarried women.
Jorge et al (2015)	Can group exercise programs improve health outcomes in pregnant women? A systematic review	SR	Pregnant women	17	QUAN	Health	Group exercise programs reduced the proportion of women on sick leave due to lumbopelvic pain.
Kai et al (2022)	Prevalence and determinants of return to work after various coronary events: meta-analysis of prospective studies	SR & MA	Working population post-coronary event	43	QUAN	Health RTW barriers	Pooled return to work prevalence was higher for men (74.9%) than women (64.4%), though this difference was not statistically significant.
Karkkainen et al (2017)	Systematic review: factors associated with return to work in burnout	SR	Working population on sick leave with burnout	10	QUAN	RTW barriers	Male gender was a significant predictor of faster and full-time return to work within 6 months. Men returned more rapidly than women, though the underlying reasons (e.g., stigma, financial pressure) were not discussed in depth by the authors.
Kausto et al (2008)	Partial sick leave – review of its use, effects and feasibility in the Nordic countries	Narrative review	Nordic working population	6	QUAN & QUAL, MM	WE RTW barriers	60–80% of partial sick leave is granted to women. Use of partial sick leave did not consistently improve return to work. Gendered occupations and education levels influenced uptake.
Körlin et al (2009)	Sickness absence among women and men in the police: A systematic literature review	SR	Police force employees	21	QUAN	WE Health	Women had higher sickness absence than men despite higher fitness. Female officers were more likely than male officers to have sickness absence due to occupational frustration and poorer work performance due to unequal access to benefits. No difference in sickness absence rates was found for male and female police in a Norwegian sample.
Kuijer et al (2006)	Prediction of sickness absence in patients with chronic low back pain	SR	Workers with chronic low back pain	17	QUAN	Health	No evidence was found that gender predicted either return to work or sickness absence.
Lancman & Barroso (2021)	Mental health: Professional rehabilitation and the return to work	SR	Workers with mental health problems	47	QUAN & QUAL	Health Work– family conflict	No findings were reported in the results, but the authors’ discussion highlighted women’s multiple roles (mother, worker, caregiver), increasing sickness absence.
Lenssinck et al (2013)	Consequences of inflammatory arthritis for workplace productivity loss and sick leave	SR	Working population with inflammatory arthritis	47	QUAN	Health	The findings were mixed when assessing gender as a predictor. One paper showed that female sex led to greater sickness absence, but another showed that female sex was protective of sickness absence for arthritis.
Leso et al (2020)	The Impact of thyroid diseases on the working life of patients	SR	Workers with thyroid disease	24	QUAN	WE RTW barriers	Female patients returned to work later and more often reported reduced workload compared to men.
Liao et al (2023)	Is there a bidirectional relationship between workplace bullying and sickness absence?	SR & MA	General working population	28	QUAN	WE	Both men and women had an increased sickness absence risk with bullying. No significant gender difference was observed.
Liedberg et al (2021)	Qualitative evidence from studies of interventions for chronic MSK pain and return to work	SR	Workers with chronic MSK pain	18	QUAL	Health	Women discussed challenges balancing home, work and pain more often. 70% of respondents discussing life balance were women.
Lin et al (2018)	Impact of lower urinary tract symptoms on work productivity in female workers: A systematic review and meta-analysis	SR & MA	Female working population with urinary tract symptoms	14	QUAN	Health	No significant difference in absenteeism was found between the lower urinary tract symptoms group and the control group. Lower urinary tract symptoms had more impact on presenteeism than absenteeism.
Linder et al (2020)	Inequalities in the economic consequences of depression and anxiety in Europe: A systematic scoping review	SR	European population with depression and anxiety	17	QUAN	Health	Anxiety was only linked to sickness absence for women in one study. Combined mental disorders (CMD) increased sickness absence for both genders. Slightly higher sickness absence risk among women, but interaction effects were not statistically significant.
Liu et al (2007)	A systematic review evaluating health-related quality of life, work impairment, and health-care costs and utilization in abnormal uterine bleeding	SR	General population with abnormal uterine bleeding (AUB)	74	QUAN	Health	20–55% of women with AUB took time off work. One study estimated 3.6 weeks less work/year for heavy bleeders.
Longo et al (2020)	Financial toxicity associated with a cancer diagnosis in publicly funded healthcare countries: A systematic review	SR	Working population with cancer	32	QUAN	Health	Most women had a single absence averaging 32.3 weeks. At 12 months, 21.6% had not returned to work. Gender not directly analysed, but findings suggest long-term sickness absence is common in female cancer survivors.
López-Faneca et al (2025)	Prognostic factors affecting return to work in cancer patients: a systematic review	SR	Working population with cancer	20	QUAN & QUAL	Health RTW barriers	Older women with breast cancer do not return to work, but other studies show no significant difference between age groups.
MacDonald et al (2024)	Physical job demands in pregnancy and associated musculoskeletal health and employment outcomes: A systematic review	SR	Pregnant and postpartum workers	16	QUAN	Health	Pregnant women are at increased risk of sickness during and after pregnancy as a result of pelvic girdle pain and low back pain. Heavy lifting, standing, and physical work are associated with pelvic girdle pain and low back pain for pregnant and postpartum women.
Mackenzie et al (2018)	Women’s experiences of pregnancy-related pelvic girdle pain: A systematic review	SR	Women with pelvic girdle pain	8	QUAL	Health	Women described frustration with workplace responses and sickness absence due to pelvic girdle pain. Coping and support needs were emphasised.
Marzan et al (2022)	SR and dose–response meta-analysis on alcohol consumption and sickness absence	SR & MA	Working population with alcohol use	21	QUAN	Other (men and women alcohol consumption habits, which in turn affect sickness absence)	Higher risk of sickness absence in both men and women with high-risk drinking. Gender differences are not statistically significant.
Mbengi et al (2016)	Barriers and opportunities for return-to-work of cancer survivors: Rapid review and expert consultation	Rapid review	Working cancer survivors	43	QUAN & QUAL	Health	Women, especially breast cancer survivors, reported worse return to work outcomes. Higher education supported a faster return to work, especially for men.
McIntosh et al (2000)	Low back pain prognosis: Structured review of the literature	Structured literature review	Populations with lower back pain	9	QUAN	Health	Mixed findings showed that being female was associated with both better and worse return to work outcomes. Women may present earlier, possibly with less severe conditions, and family roles may hinder recovery.
McIntyre et al (2011)	Depression in the workforce: The intermediary effect of medical comorbidity	Structured narrative review	Working-age population with depression	Not reported	QUAN	Health	The authors discuss gender differences in work stress and depression, which may, in turn, impact sickness absence rates.
Mehnert (2011)	Employment and work-related issues in cancer survivors	SR	Working-aged cancer survivors	64	QUAN	Health	Cancer affects men and women differently, and there are also major biological differences in which cancers the genders may acquire. As a result, these gender differences in cancer may impact return to work rates differently for men and women.
Merkus et al (2012)	The association between shift work and sick leave: A systematic review	SR	Working population in shift work	24	QUAN	WE	Strong evidence for an association between fixed evening shifts and sickness absence among female healthcare workers. Management support may be less available in evenings, influencing female sickness absence more than male.
Michie & Williams (2003)	Reducing work-related psychological ill health and sickness absence	Systematic literature review	General working population	49	QUAN	WE	Reduced job control was linked to increased depression and absenteeism in men, not women. The authors suggest men may respond differently to adverse work environments.
Miller & Chung (2023)	Determinants of return to activity and work after carpal tunnel release	SR & MA	Workers post-CTR surgery	48	QUAN	Health	Women returned to work significantly faster than men post-surgery.
Mullerpatan & Jadhav (2025)	Trends in work-absenteeism and return-to-work among people with spine pain in middle income countries: A need for evidence	SR	Working age people with spinal pain from low to middle income countries	3	QUAN	Health	Men had higher absenteeism than women with spinal pain.
Nandi et al (2018)	The Impact of Parental and Medical Leave Policies on Socioeconomic and Health Outcomes in OECD Countries	SR	Working parents in OECD countries	109	QUAN	Work– family conflict WE	Leave policies affect women disproportionately; longer parental leave may protect against the “motherhood penalty” but may also deter short-term labour force participation. Some policies aim to reduce discrimination against women of childbearing age.
Ndjaboue et al (2012)	Organisational justice and mental health: a systematic review of prospective studies	SR	General working population with mental health concerns	11	QUAN	WE	Both men and women are more likely to take sick leave when relational justice is lacking. Relational fairness in the workplace reduces sickness absence.
Neovius et al (2009)	Obesity status and sick leave: A systematic review	SR	General working population with BMI data	40	QUAN	Health	Some studies found that obese women had more long-term sickness absence than “normal weight” workers.
Nielsen et al (2016)	Workplace bullying and sickness absence: a systematic review and meta-analysis	SR & MA	General working population	17	QUAN	WE	Bullying increased sickness absence for both genders; three studies showed stronger effects among women.
Nilsen et al (2017)	Examination of the double burden: A systematic review of work–family conflict and sickness absence	SR	General working population	8	QUAN	Work– family conflict	Moderate evidence supports women reporting more work–family conflict and sickness absence. Stronger associations for single mothers and women with caregiving roles were reported. However, gender moderation evidence is mixed.
Nowrouzi-Kia et al (2024)	Remote work transition amidst COVID-19: Impacts on presenteeism, absenteeism, and worker well-being-A scoping review	Scoping review	General working population	8	QUAN & QUAL	WE	Women reported more distractions whilst working at home, specifically related to childcare duties, compared to men. The presence of children at home was linked to increased psychological distress and absenteeism.
Nyberg et al (2021)	Workplace violence and health in human service industries: A systematic review	SR	Health, social and education service workers	28	QUAN	WE	Psychological violence is strongly associated with sickness absence. One study assessed gender-based violence and sickness absence but found no significant association.
Parsons et al (2022)	Developing and testing a case-management intervention to support the return to work of health care workers with common mental health disorders	A mixed-methods study including a systematic review component	Healthcare workers with common mental health disorders	20	QUAN & QUAL	Health WE RTW barriers	Common mental disorder-related sickness absence is a significant burden in healthcare. Return to work is less likely after longer absences. Gender not explicitly discussed, but a female-majority workforce noted. Poor quality and heterogeneity in existing common mental disorders studies.
Pedersen et al (2018)	Systematic review of interventions targeting sickness absence among pregnant women in healthcare settings and workplaces	SR	Working pregnant women in healthcare	5	QUAN	Health WE	A 12-week exercise intervention reduced sickness absence significantly in one study. Other interventions ineffective. No workplace-specific interventions. Sickness absence is influenced by work strain and physical discomfort.
Pennick & Young (2007)	Interventions for preventing and treating pelvic and back pain in pregnancy	SR	Pregnant women with back and pelvic pain	8	QUAN	Health WE	Acupuncture and exercise reduced pain; water gymnastics lowered absenteeism (12.9% vs. 21.7%).
Peters et al (2016)	Prognostic factors for return-to-work following surgery for carpal tunnel syndrome	SR	General working-age population with carpal tunnel syndrome	11	QUAN	Health WE	Mixed findings show some studies reported slower return to work for women, while others found faster return to work. Ergonomic exposure, psychological workload and decision latitude predicted delayed return to work among women.
Pizarro & Gartzia (2024)	Paternity leave: A systematic review and directions for research	SR	Parents (fathers, mothers) on parental leave	154	QUAN & QUAL	WE	Men’s use of paternal leave was not directly associated with women’s sickness absence, unless fathers took more time off than the paternity leave allocated to them.
Politis et al (2024)	The global burden of absenteeism related to COVID-19 vaccine side effects among healthcare workers: A systematic review and meta-analysis	SR & MA	Healthcare workers	4	QUAN	Health WE	After receiving the COVID-19 vaccination, women had more absenteeism than men.
Probst et al (2018)	Implementation, mechanisms and effects of maternity protection legislation	Realist narrative review	Pregnant workers	42	QUAN & QUAL	Health WE	Limited pregnancy work accommodations may increase sickness absence. Pregnant workers sometimes use sick leave instead of maternity leave due to system incentives. Sickness absence is viewed as a consequence of poor workplace adaptation. Gender inequality is linked to workplace norms and maternity protection legislation implementation failures.
Richardson et al (2017)	Office design and health: a systematic review	SR	General working population	15	QUAN	WE	Working in large open-plan offices is associated with long-term sickness absence, but this was significant for women only. Open office plans are linked to stress and dissatisfaction.
Russo et al (2024)	Prognostic factors for return to work in patients affected by chronic low back pain: a systematic review	SR	Working age population with chronic low back pain on sick leave	20	QUAN	Health RTW barriers	Being a woman was associated with delayed return to work and longer sickness absence.
Sabariego et al (2018)	Effectiveness of integration and re-integration into work strategies for persons with chronic conditions	SR	Working-age adults with chronic conditions	21	QUAN	RTW barriers	The “30-60-90-day rule” in Finland improved return to work outcomes, especially for women.
Safwan et al (2024)	Menopause in the workplace: Challenges, impact, and next steps	Narrative review	Working-age women experiencing menopause	Not stated	QUAN	Health	Menopause symptoms such as hot flashes, fatigue, and sleep issues are associated with reduced productivity and increased sickness absence. A large US study found 57% increase in productivity loss due to untreated symptoms.
Sagherian et al (2019)	Fatigue and risk of sickness absence in the working population: A systematic review and meta-analysis	SR & MA	General working population	16	QUAN	Health	Fatigue is significantly associated with long-term sickness absence, and there was a stronger pooled effect in men than women, but not significant. Women reported higher fatigue levels, but male fatigue was more predictive of sickness absence.
Santos et al (2023)	Prevention of low back and pelvic girdle pain during pregnancy: A systematic review and meta-analysis	SR & MA	Pregnant women with/without low back and pelvic girdle pain	6	QUAN	Health	Exercise interventions did not significantly reduce sickness absence due to low back and pelvic girdle pain.
Schmier et al (2006)	Cost of obesity in the workplace	SR	Working population who are obese/overweight	Not reported	QUAN	Health	Obese Swedish women aged 30–59 took 1.5x more sick leave than the general population.
Schou & Moan (2016)	Alcohol use-sickness absence association and the moderating role of gender and socioeconomic status	Literature review	Working population reporting alcohol use and sickness absence	28	QUAN	Health	Women showed stronger associations between alcohol use and sickness absence in some studies. Binge drinking and alcohol-related sickness absence were more prevalent among low-income women.
Sedeh et al (2022)	Prevalence, risk factors, and prevention of occupational contact dermatitis among professional cleaners: a systematic review	SR	Professional cleaners	39	QUAN	WE	Female cleaners reporting with hand rashes were more likely to be absent from work (31%) than builders (5%), though not statistically significant.
Sharpe et al (2022)	Return-to-work after work-related injury in the construction sector: A scoping review	Scoping review	Injured construction workers	22	QUAN & QUAL	Attitudes & norms Health	One study found that being female predicted delayed return to work following back injury. The authors discuss that male-dominated cultures in construction promote presenteeism. Additionally, women may delay their return to work until fully recovered.
Shi et al (2014)	A systematic review of prognostic factors for return to work following work-related traumatic hand injury	SR	Workers with hand injuries	8	QUAN	RTW barriers	Three studies examined gender effects on return to work - none found statistically significant gender differences.
Slebus et al (2007)	Prognostic factors for work ability in sick-listed employees with chronic diseases	SR	Sick-listed employees with chronic conditions	6	QUAN	Health RTW barriers	Being male and being the breadwinner were associated with return to work after myocardial infarction and chronic low back pain. Being female was a risk factor for non-return to work.
Soliman et al (2016)	The direct and indirect costs associated with endometriosis: A systematic literature review	SR	Population with endometriosis	12	QUAN	Health	Endometriosis leads to significant productivity losses (e.g., €3,314 per person/year in Austria). As a female-only condition, it may partially explain higher sickness absence among women.
Steenstra et al (2005)	Prognostic factors for duration of sick leave in patients sick-listed with acute low back pain: A systematic review of the literature	SR	Workers with acute low back pain	18	QUAN	Health	Women with acute low back pain were more likely to have prolonged sickness absence. The authors suggest biological and/or psychosocial explanations (e.g., caregiving roles, physician perceptions).
Steenstra et al (2017)	Systematic review of prognostic factors for return to work in workers with sub acute and chronic low back pain	SR	Workers with sub-acute/chronic low back pain	22	QUAN	Health WE	No consistent gender association found in sub-acute cases. Mixed findings for chronic low back pain: some studies showed men return to work earlier, others showed no difference.
Sterud et al (2018)	A systematic review of working conditions and occupational health among immigrants in Europe and Canada	SR	Immigrant workers	82	QUAN	Health	Non-Western immigrant women had higher rates of both general and pregnancy-related sickness absence in Norway and Sweden.
Stovner et al (2006)	Epidemiology of headache in Europe	Narrative epidemiological review	General European population	Not specified	QUAN	Health	Women had higher rates of headache/migraine (up to 83% vs. 69% in men). Some evidence links migraines to increased sickness absence, suggesting an indirect gender difference in SA rates.
Street & Lacey (2015)	A systematic review of studies identifying predictors of poor return to work outcomes following workplace injury	SR	Workers with workplace injuries	9	QUAN	RTW barriers	Being female was consistently associated with poor return to work outcomes. Potential mechanisms include caregiving burden, lack of support, and household responsibilities (e.g., dependents).
Stuge et al (2003)	Physical therapy for pregnancy-related low back and pelvic pain: A systematic review	SR	Pregnant women with low back and pelvic pain	9	QUAN	Health	Water gymnastics and physical therapy were linked to reduced sickness absence in late pregnancy and postpartum.
Troelstra et al (2020)	Smoking and sickness absence: A systematic review and meta-analysis	SR & MA	Working population who smoke	43	QUAN	Health	Smoking increases sickness absence, but no gender differences were found in the smoking–sickness absence association.
Truchon & Fillion (2000)	Biopsychosocial determinants of chronic disability and low-back pain: A review	Critical literature review	Individuals with low back and pelvic pain and chronic disability	18	QUAN	Health	Mixed findings: men associated with longer sickness absence, women with non-return to work. Gender may not be a strong predictor overall; compensation bias may influence male sickness absence duration.
van Benten et al (2014)	Recommendations for physical therapists on the treatment of lumbopelvic pain during pregnancy: a systematic review	SR	Pregnant women with lumbopelvic pain	22	QUAN	Health	Physical therapy reduced pain and sickness absence during pregnancy. Exercise therapy showed better outcomes than standard antenatal care. Effective treatment may prevent sickness absence.
Van Leemput et al (2022)	Return to work after primary total knee arthroplasty under the age of 65 years: A systematic review	SR	Working-aged adults post-Knee Arthroplasty	14	QUAN & QUAL	Health	Women had longer return to work periods post-surgery and more sickness absence pre-surgery. One study reported lower return to work rates for women (83%) vs. men (88%).
Vander Weerdt et al (2023)	Is paid sick leave bad for business? A systematic review	SR	Working population	43	QUAN	WE Attitudes & norms	Women, particularly low-wage workers, showed increased use of paid sick leave. One study found a significant increase in absences only among women after paid sick leave was implemented.
Verdonk et al (2022)	Menopause and work: A narrative literature review about menopause, work and health	Narrative review	Working women experiencing menopause	36	QUAN & QUAL	Health	Menopausal symptoms were linked to higher sickness absence and presenteeism. Women may underreport symptoms; flexible work policies and open dialogue are recommended to address menopause-related sickness absence.
Wade (2011)	The societal costs of insomnia	SR	The general population with insomnia	49	Not specified	Health	Insomnia was associated with increased sickness absence, especially among women and blue-collar workers.
Wahlstrom & Alexanderson (2004)	Physicians’ sick-listing practices	SR	Physicians and the working population	28	QUAN & QUAL	Attitudes & norms	Female patients were more likely to receive part-time sick leave compared to male patients. Male physicians rated male patients as more impaired. Physician and patient gender influenced sickness absence decisions.
Wegrzynek et al (2018)	Tertiary return to work interventions for chronic pain: A systematic literature review	SR	Workers with chronic pain	16	QUAN	RTW barriers	Women had worse return to work prognosis but benefited more from multidisciplinary interventions (e.g., CBT + physiotherapy).
Weng et al (2013)	Smoking and absence from work: systematic review and meta-analysis of occupational studies	SR & MA	General working population	29	QUAN	Health	Male smokers had more sick leave days than male non-smokers, whereas for women, the difference was smaller and not statistically significant.
Xia et al (2025)	Bariatric surgery, employment, and productivity outcomes: A systematic review	SR	Working adults with obesity who had bariatric surgery	42	QUAN	Health	No gender difference between rates of sickness absence following surgery, but both men and women had significantly higher rates compared to the controls.

### Reviews with a focus on sickness absence gender differences

In total, 12 of 133 included reviews focused specifically on gender differences in sickness absence or explicitly examined one or more a-priori hypotheses (table 3) (28–39). Of these, 7 examined health impacts on sickness absence, mostly women’s reproductive health, including: pregnancy and menopause (31, 33); breast cancer and work (28, 30); menopause (39) and mental illness (36). Work factors and sickness absence were examined in 2 reviews (33, 35), while 1 review investigated the work–family conflict hypothesis (37). Several explored more than one hypothesis.

**Table 3:** Categorisation of 12 included systematic reviews and meta-analyses where the primary focus was gender differences in sickness absence.

Reference	Work–family conflict	Work environment	Health	Barriers to return-to-work	Attitudes and norms	Health beliefs
Banning (28)		✓	✓	✓		✓
Bekker et al (29)		✓	✓		✓	
Bijker et al (30)		✓	✓			
Brunner et al (31)		✓	✓			✓
Gjellestad et al (32)			✓			
Henrotin & Gulisano (33)		✓	✓			
Hensing et al (34)			✓			
Kausto et al (35)		✓		✓		
Körlin et al (36)		✓				
Nilsen et al (37)	✓					
Schou & Moan (38)			✓			
Verdonk et al (39)			✓			

The majority (N=11/12) included no information about how sickness absence was defined or measured, and if measured differently by studies included in the same review (37). Most common methods involved employer or national social insurance registries, but self-report studies were included. Under half (N=5) presented any quality assessment, but those that did reported mixed quality. For topics on breast cancer, authors deemed studies to be of high quality (28, 30). Whereas for reviews examining sickness absence and pregnancy, alcohol use or work–family conflict, the study quality was mixed, with most scoring either moderate or low (33, 37, 38).

### Can gender differences in work–family conflict explain sickness absence gender differences?

Eight reviews investigated the work–family conflict hypothesis but together found only limited evidence to support it as a cause (29, 31, 33, 34, 37, 40–42). Reviewers highlighted study limitations, including measuring work–family conflicts at only one time point (37).

One review (37) found evidence of a moderate association between a range of work–family conflict measures and sickness absence among women. However, study findings were inconsistent, with others reporting no associations or associations stronger for men (37, 34). Generally, women scored significantly higher for perceived work–family conflict and family–work conflict compared to men, perhaps reflecting gender-different experiences of work–family conflict, which could affect sickness absence.

None of the hypotheses – pregnant women would have more work interruptions if they had several children (31); women may experience additional stress because of their multiple roles (work and home) (42); the combination of work–family conflict and health issues could influence long-term sickness absence; or the greater domestic responsibilities borne by women could limit their ability to fully rest and recover whilst sick leading to longer sickness absence spells compared to men (40) – were supported by evidence.

### Can gender differences in health problems explain sickness absence gender differences?

Most reviews focused on health and sickness absence (N=94), generally with mixed findings (28–34, 36, 38–40, 42–124). Both genders took more sick leave when they had health problems than when they did not. A range of health issues were studied including common mental disorders, musculoskeletal conditions, obesity and overweight, chronic diseases, long-term disabilities, unhealthy behaviors (ie, alcohol and smoking), and neurological conditions. Mostly, reviews focused on the health condition and reported sickness absence as a secondary outcome, stratified by gender, without additional exploration or discussion.

Mental health was often examined as a risk factor for sickness absence rather than a potential explainer of gender differences. Most reviews reported mental illness was associated with sickness absence and that mental illness was more common among women than men (34, 42) but did not examine whether the excess prevalence explained gender differences. One review found men who disclosed mental illness were more likely to take sick leave than women (66), and another found no differences between sickness absence rates among men and women with anxiety or depression (90). Alcohol use was a risk factor for sickness absence in both genders (38, 95), but one review found stronger associations between number of alcoholic drinks and binge-drinking, and number of sickness absence days among women compared to men.

Women’s reproductive health and sickness absence were the subject of several reviews, particularly pregnancy. It was postulated that women may be more likely to take sick leave when pregnant than when not pregnant to protect their health and that of their unborn child (31). Another putative explanation was that hormonal changes might impact work participation during physiological menstruation and were exacerbated by menstrual disorders (eg endometriosis) (32). Two reviews suggested higher rates of sickness absence and lower work productivity among women experiencing menopausal symptoms (39, 107). Two reviews reported evidence that endometriosis was associated with higher rates of sickness absence and unemployment (32, 113).

### Can gender differences in the work environment explain sickness absence gender differences?

Overall, 45 reviews explored gendered work factors and sickness absence. There was evidence that both genders experienced work adversities (including bullying, aggression, sexual violence/harassment and relational injustice), which were associated with sickness absence (28–31, 33, 35, 36, 41, 43, 51, 58, 68, 71, 72, 75, 80, 81, 87, 100, 103–106, 114, 125–143). One review (135) reported higher rates of bullying among female versus male workers and found an association between bullying and risk of sick leave but did not investigate whether the bullying explained their higher rates of sickness absence. Regarding other workplace adversities, workplace discrimination (perceived as being found less capable) (133), poorer career prospects and opportunities for role mobility were identified among female workers, each of which were associated with sickness absence, but the directionality of these associations was unclear and lengthy sickness absence could directly influence career opportunities for women.

Work-related factors such as physical fitness were measured in relation to sickness absence in one study of police (36). The study found that women generally had higher rates of sickness absence than men, but had higher fitness scores, and fitness score predicted sick leave among men but not women. The authors suggested the historic, masculine nature of the police force and possible tokenism might cause gender differences in sickness absence. Sexual harassment and unequal promotion prospects were associated with worse subjective health, and policewomen were more likely than men to take sickness absence due to career frustrations. Among female blue-collar workers, women had longer durations of sickness absence from musculoskeletal disorders and trauma (68). Mechanisms were not explored, but the reviewers suggested elements of male-dominated environments, such as poor-fitting uniforms unsuitable for female anthropometry and harassment, could contribute.

One review found pregnant women had more sickness absences in workplaces with a higher proportion of female employees compared to workplaces with a higher proportion of males (31).

### Can gender differences in attitudes and norms around sickness absence explain gender differences in sickness absence?

Seven reviews explored whether gender differences in attitudes and norms affected sickness absence duration, with the role of the ‘male provider’ considered in several (29, 75, 111, 143–146). Reviewing qualitative evidence (144) showed that men were often the main financial provider, potentially influencing their views about time to return-to-work. However, no evidence was provided that this impacted sickness absence rates.

Beliefs and attitudes among men about work were thought to play a role based on perceived work ethic. It was hypothesized taking sick leave might be perceived as a lack of work ethic, incentivizing male workers to ignore work injuries, especially in male-dominated work environments. One review found male physicians viewed sick-listed male patients as more severely impaired than female patients, whilst female physicians considered men and women equally work impaired or women were more impaired (146). However, the medical indications for sick leave were unreported, which may influence the findings.

### Can gender differences in health beliefs about sickness absence explain gender differences in sickness absence?

Two reviews explored the role of health beliefs on sickness absence to explore gender differences (28, 31). Among breast cancer survivors, women felt returning to work following sickness absence was good for their health, and holding this belief encouraged women to go back to work faster (28).

### Can gender differences in barriers to return-to-work explain sickness absence gender differences?

Prolonged sickness absence can be caused by barriers to successful return-to-work after illness (28, 35, 51, 84, 87, 96, 103, 112, 134, 147–153). Overall, reviews which reported on the duration of sickness absence found men returned to work faster than women. The evidence does not suggest men recover faster, but rather there may be differential barriers to returning between genders. Examining return after a workplace injury, one review found female gender was a risk factor for poor return-to-work outcomes (134, 151). The authors suggested domestic responsibilities prevented women from giving full attention to recovery. Another explanation may be levels of support provided for return-to-work: after breast cancer treatment, women reported wanting to return for a sense of structure and belongingness, but appropriate adjustments were not provided, and some women met with hostility on their return (28). Men were more likely to be offered vocational rehabilitation (VR) than women according to one review (134), however the industries to which workers were returning were unreported, an important omission, given VR is more often available for white-collar male-dominated industries. Examining partial sick leave as an intervention to facilitate rates of return-to-work, a review found most recipients were women (35).

### Other hypotheses

Few included reviews could not be categorized by the pre-defined framework. However, some reviews explored gender alongside other demographic factors, adopting an intersectional approach (31, 56, 73). For example, race differences were found among women taking sick leave following a breast cancer diagnosis, such that 44.4% of black women took sick leave compared to 25.8% of white women (25.8%) (56). However, there were probably considerable confounders caused by inequalities in diagnoses and treatment. Higher educational attainment was found to be associated with increased sickness absence among women (31, 73). However, educational attainment is importantly associated with occupation, with likely effects on sickness absence rates. This intersectionality was also highlighted in a review showing the relationship between diagnosed alcohol misuse and duration of sickness absence was greater among women with lower incomes (38).

### Trends in research

Our umbrella review revealed some chronological changes in the sickness absence literature (figure 2). Between 2000 and 2006, most reviews focused on health issues in the workforce, specifically back pain and mental illness in relation to outcomes including sickness absence and its economic consequences. Reviews published from 2007 focused more on the impact and experiences of workers, and from 2009 started to report gender disparities. Return-to-work reviews were more prevalent in 2015 and 2019, focusing on the proportion of employees on sick leave who returned to work and the duration of sickness absence. Previously under-researched topics including menopause, women’s reproductive health (eg, endometriosis) and paternity leave, started to be published from 2022.

**Figure 2:**
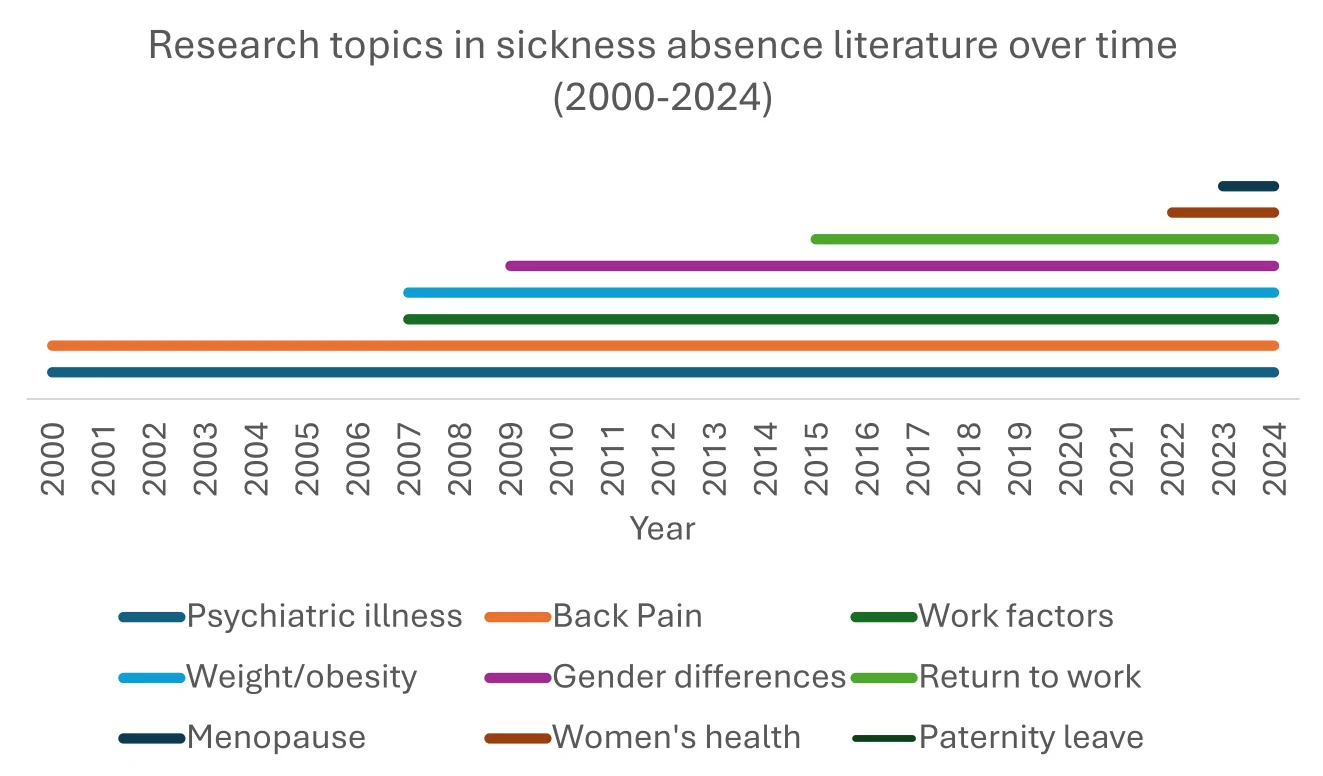
Changing patterns of sickness absence literature over time (2000–2024).

## Discussion

This umbrella review identified 133 eligible reviews, only 12 (9.5%) of which specifically focused on gender differences in sick absence. There was a major emphasis on health (N=94) or work environment factors (N=45) for review topics. In contrast, other hypotheses: the role of work–family conflict (N=8); attitudes and norms around sickness absence (N=7); support to return to work after illness (N=16); and beliefs about health (N=2) were understudied. Only around half of the reviews (N=68) published a quality assessment of their included papers. When quality assessment was reported, papers were mostly rated as medium- or low-quality. Although there were some better-rated papers in specific areas, this finding emphasizes a need for better quality research on this topic. Overall, despite an extensive number of relevant reviews, the underlying reasons for gender differences in sickness absence remain inadequately explained.

The literature focused primarily on the role of health, particularly women’s health issues (31, 33, 39), as a cause of gender differences in sickness absence. However, they did not provide compelling evidence that women’s health alone explained the observed gender differences. Where sickness absence was explored across a wider range of health conditions, it was often reported as a secondary outcome, leaving reasons for differences unexplored. A surprising lack of reviews focused on sickness absence for conditions that are exclusive to, or more prevalent among, men such as prostate cancer or cardiovascular disease. It is plausible that gender differences might be attributable to male behaviors and beliefs and, for example, men may underutilize sickness absence because of established stigmas or societal norms, leading to presenteeism, with possible consequences for long-term health (154). Men may be more likely to normalize or ignore injuries at work to improve their perceived credibility or in the belief that taking sickness absence detracts from their masculinity and role as the financial provider (155). Researchers using case studies of men and women experiencing depression found men were less likely than women to view sickness absence as reasonable (22). However, another study found no gender differences in attitudes to sickness absence but found increased tolerance for sickness absence in gender-dominated fields compared to gender-integrated occupations (156). Traditional gender roles might also influence behaviors: women who are traditionally expected to adopt caregiving responsibilities may experience lower levels of perceived and internalized stigma when taking sickness absence for a similar state of health (22). It should be acknowledged that ‘sex’ and ‘gender’ are often used interchangeably in literature, despite referring to distinct biological and sociocultural constructs (25). Future research should explicitly differentiate between these concepts, as comparing hypotheses based on sex with those based on gender could provide important theoretical insights and improve understanding of the mechanisms underlying gender differences in sickness absence.

Given the vast body of literature examining gender in the workplace, we anticipated the included reviews would provide a clearer narrative on how work factors influence gender differences in sickness absence. There was evidence that, compared with men, women were more likely to experience workplace adversities including discrimination, harassment and bullying (135), but not that this differentially affected sickness absence; the risk of which was increased by adversities in both genders (29, 125, 126, 129). It was hypothesized that there may be more sickness absence among women in male-dominated workplaces (36), possibly due to a lack of adjustments accommodating women’s health, but a review of pregnancy-related sickness absence found that absence rates among pregnant employees in female-dominated workplaces were higher than in male-dominated workplaces (31). Taken together, the body of reviews suggested a possible causative effect for a range of workplace factors (policies, physical work demands, psychosocial demands, perceptions about behaviors) but failed to provide clear-cut evidence in support of any.

Our review shows that hitherto, research has focused on observing gendered exposures to risk factors for taking sick leave, laying a broad but fragmented foundation. Consequently, an abundant list of risk factors exists, but without explanatory analysis to understand why these factors cause gender differences in sickness absence. Additionally, investigation of individual single risk factors may be too simplistic if, as is more likely, there is a complex interplay of multiple factors which contribute to gender differences in sickness absence. That women have specific health conditions different from men may be part of the story (although not yet established as a cause) but how they behave in response to symptoms and illness, what healthcare they seek and whether or not they take sick leave are likely to be influenced by their own health beliefs, the culture, beliefs and norms of their community at home and at work. These factors together will influence whether they are well enough to go to work. Clearly, a multi-faceted approach is needed to assess the intersectionality of multiple factors or hypotheses to produce a deeper understanding of the complexities involved and enable implementation of effective prevention strategies.

### Strengths and limitations of this umbrella review

A broad search strategy was adopted to capture evidence about the team’s a-priori hypotheses, as well as to identify lesser-known hypotheses. Consequently, approximately 90% of the included reviews were not primarily focused on the gender difference in sickness absence, and sickness absence was frequently measured as a secondary outcome. Few included studies started with an a-priori hypothesis, so reviews were synthesized according to risk factors interpreted through the pre-defined framework. It is feasible the categorization of reviews may have limited our interpretation of review findings. Further, nearly half of the included reviews did not use (or report) a formal quality assessment tool, so it is difficult to ascertain from this umbrella review the quality of evidence.

Despite using a comprehensive search strategy of seven databases, applying no date restriction and including research published in several languages, it is possible we have not captured all relevant reviews. Additionally, it is plausible the search strategy may have captured more reviews on women’s than men’s health because differences in sickness absence are more generally believed to be associated with women’s health, so researchers may be more likely to use the term “sickness absence” in the title/abstract. If so, this highlights a challenge that will need novel approaches from reviewers in future. Further, the inclusion criteria included all types of reviews (eg, narrative reviews, meta-analyses, systematic reviews) in the umbrella review. The heterogeneity of methodological approaches used across review types is acknowledged as a limitation but the aim was to provide a comprehensive overview of all existing evidence that may contribute to hypotheses of gender differences in sickness absence; therefore, inclusion of all review findings was deemed necessary. Similarly, to ensure we included as many relevant hypotheses as possible, reviews on specific women’s health issues, eg pregnancy, were eligible for inclusion as they met the criteria of having an exposure involving gender without a male comparison, which may also have introduced or exacerbated any such bias.

AI was used to screen reviews for eligibility and some basic data extraction. Previous studies have shown high precision at the screening stage for systematic reviews (157) (95%), outperforming human reviewers. However, there is less evidence for the use of AI in umbrella reviews. It is plausible AI might have wrongly rejected reviews, increasing the risk of potential bias. However, at each stage, at least one member of the research team screened all reviews for eligibility to mitigate this risk. Similarly, all included reviews underwent data extraction by researchers, and ‘false positives’ would have been identified at this stage.

The umbrella review highlighted other methodological shortcomings. Methods to measure sickness absence varied and were often inadequately described or critiqued by reviewers. Few reviews examined longitudinal trends in sickness absence. While unexplained currently, exploring temporal patterns may allow us to identify broader cultural shifts, such as changing attitudes towards pregnancy and work, or the medicalization of pregnancy-related symptoms. Additionally, examining sickness absence longitudinally across the life course will enable insights into age-period-cohort effects which may influence (eg menopause) sickness absence.

We found disappointing levels of evidence supporting the common hypotheses used to explain gender differences in sickness absence. Some key priorities for future research include exploring the double burden for women (ie, homemaker responsibilities versus work demands). Although measurement of work–family conflict might partly capture this construct, there were limited reviews on this topic with inconsistent findings. Recent primary studies not yet captured in systematic reviews suggest some associations between work–family conflict and sickness absence, but do not currently provide evidence suggesting differential effects by gender (158, 159). It is plausible current methods to measure work–family conflict are inadequate, and measurement once (37) or only in one direction (ie, work–family conflict and family–work conflict) is masking an effect. It is also possible existing measures capture only practical burdens, but not psychological burden which may impact genders differently. Future studies should aim to comprehensively measure all facets of work–family/family–work conflict using reliable, longitudinal measures (37).

Our review identified a significant gap in the role of health beliefs and gender inequalities on sickness absence (160–165). For instance, variations may exist in taking sick leave to mitigate personal occupational hazards, subjective assessments of work capability, and/or perceived symptom severity. Studies could usefully explore gender differences in health beliefs and intentions of individuals who are considering taking sick leave, currently on sick leave or in the process of returning to work. Another important aspect would be to capture gender differences in motivations to (re-)enter the labor market. Understanding these could provide valuable insights into attitudes towards sickness absence and its underlying determinants.

### Concluding remarks

There is currently insufficient evidence to explain gender differences in sickness absence. Despite numerous hypotheses, many of which have strong face validity, there was limited rigorous evidence to underpin these. Partly, this can be explained by the fact that gender differences in sickness absence were addressed only tangentially in many identified reviews. Our findings suggest researchers need to take a cautious approach to avoid conflating plausible hypotheses with established explanatory evidence.

We also found important biases in the evidence, with much of it focused on women-specific health factors (166), including pregnancy and menopause, and limited attention on men and their health and work, effectively preventing a full picture of how gender influences sickness absence. Together, these findings highlight the need for a more focused and theoretically informed research agenda which moves beyond the identification of gendered risk factors, implementing an exploratory approach to causality to robustly investigate the mechanisms by which gender affects sickness absence.

## Supplementary material

Supplementary material 1 Supplementary material
