# Supplementary material for: A review of reviews exploring gender differences in sickness absence
Source: Scand J Work Environ Health. 2026 Oct 26;52(6):633–60. doi: 10.5271/sjweh.4321 (PMC13732786; doi:10.5271/sjweh.4321)
Supplement: Supplementary file 1 [file SJWEH-52-6-633-s001.pdf]

A review of reviews exploring gender differences in sickness absence[1]

by Amber R McKenzie, PhD, Bethany Croak, PhD, Lise Gulli Brokjøb, BSc, Sharon AM Stevelink, PhD, Ira Madan, MD, Beate Brinchmann, PhD, Wendy Nilsen, PhD, Karen Walker-Bone, PhD, Nils Abel Aars, PhD, Arnstein Mykletun, PhD[2]

1. Appendices
2. Correspondence to: Arnstein Mykletun, PhD, Department of Community Medicine, University of Tromsø, Norway. [E-mail: arnstein.mykletun@uit.no]

Appendix A. Example of the database search string used in Embase (Ovid)

**1**	**exp absenteeism/**	**22374**
**2**	exp medical leave/	9292
**3**	(sick* absence* or sick* leave or disab* leave or sick* day* or ill* day* or work* absence* or disab* benefit* or disab* allowance*).mp. [mp=title, abstract, heading word, drug trade name, original title, device manufacturer, drug manufacturer, device trade name, keyword heading word, floating subheading word, candidate term word]	15987
**4**	1 or 2 or 3	35898
**5**	exp gender/ or exp "gender and sex"/ or exp gender inequality/ or exp "discrimination against sexual and gender minorities"/	1327502
**6**	exp female/	12851239
**7**	exp male/	12539931
**8**	(gender* or sex or wom*n or men or man or male* or female*).mp. [mp=title, abstract, heading word, drug trade name, original title, device manufacturer, drug manufacturer, device trade name, keyword heading word, floating subheading word, candidate term word]	17700593
**9**	5 or 6 or 7 or 8	17846196
**10**	exp "review"/ or exp "systematic review"/	3357128
**11**	exp meta analysis/	326996
**12**	(systematic review* or scoping review* or literature review* or meta$analys* or umbrella review*).mp. [mp=title, abstract, heading word, drug trade name, original title, device manufacturer, drug manufacturer, device trade name, keyword heading word, floating subheading word, candidate term word]	788939
**13**	10 or 11 or 12	3672463
**14**	4 and 9 and 13	1088

Appendix B. Methodology for use of AI in screening, data extraction and risk of bias assessment

Artificial intelligence (AI) has been researched to support the methodology process of identifying and screening relevant articles. Before using the AI in the screening and extraction phase, the platform was trained. To do this, the reviewer provided the platform with the research question and asked the platform to provide a summarised response to assess the platform's accuracy and prior knowledge on the topic. Once this was established, the reviewer provided ChatGPT with the inclusion criteria and example reviews, and the reviewer asked the platform if the example reviews were eligible for inclusion. Once ChatGPT had correctly identified the eligible reviews, we moved on to title and abstract screening with AI. Several steps were conducted to complete the screening phase.

Step one: screen title/abstract

During the abstract and title screening phase, the reviewer provided ChatGPT with information including the umbrella review objectives and protocol. Each review title and abstract were first provided to ChatGPT by the reviewer who asked if the article was eligible for inclusion; the first 10% of articles screened by AI were checked by one reviewer for interrater reliability. The reviewer then independently screened all titles and abstracts at this stage. If any discrepancies were identified between ChatGPT and the reviewer, the final decision on inclusion was made by the reviewer. A second reviewer independently reviewed 25% of all abstracts and titles selected at random to further assess interrater reliability.

Step two: full-text screening

The reviewers also used ChatGPT in the full-text screening process. The reviewers provided ChatGPT with the full-written text of each review and requested ChatGPT to identify whether the article was eligible for inclusion in the umbrella review. As with the abstract and title screening stage, 10% of articles screened by AI were checked by the reviewer. A second reviewer independently reviewed all articles at full-text screening. Any discrepancies between the reviewers and ChatGPT were discussed and resolved between the initial two reviewers; however, the human reviewer's final decision was always selected over ChatGPT. If consensus was not achieved, the wider research team were consulted. Compared with AI, the inter-rater agreement was 76%, with a moderate Cohen's Kappa of 0.53. Almost all disagreements were flagged where ChatGPT included reviews where a consensus to exclude was made by the human reviewers (n=40). Two reviews were included by ChatGPT but excluded by the reviewers; however, a final consensus was made by the reviewers to include the reviews. The full screening process is reported in a PRISMA flow chart (Figure 2). Reasons for exclusion at full-text screening stage were noted.

After the full-text screening phase, all eligible systematic reviews and meta-analyses underwent quality assessment. The Critical Appraisal Skills Programme (CASP) Systematic Review Checklist was used to assess the quality of each review. The quality assessment was carried out by a human reviewer for all articles and supported by ChatGPT, where the reviewer provided the CASP questions and the full article to the AI platform to complete the quality assessment. A second human reviewer independently assessed the quality of 10% of the articles to assess interrater reliability. Reviews were not excluded based on quality but instead we report on the current quality of existing literature on this topic.

Step three: Data extraction

Data from the included reviews and meta-analyses were extracted using a data extraction form developed specifically. The data extraction form was designed in Microsoft Excel following guidance provided by the JBI methodology for umbrella reviews with adaptations to reflect the specific aims of the current umbrella review. The reviewer piloted the data extraction with n=3 full-text articles and outcomes were discussed with the wider team. Data extracted from the reviews using ChatGPT included title, author and year of publication, review objectives, review type, sample details, number of databases sourced and searched, date of database search, publication date range of studies included in reviews, number of studies per review, country of origin for each study, quality assessment measure used, types of studies including in the review, methods used for synthesis, relevant hypothesis for gender differences in sickness absence (i.e., work environment, work-family conflict, health, attitudes and norms, health beliefs, barriers to return-to-work or other hypotheses), main findings related to sickness absence, prevalence of sickness absence for overall sample by gender/sex, main findings related to gender differences for sickness absence, and limitations declared by the original authors or identified by the current research team. ChatGPT was also employed at this phase. The reviewer instructed ChatGPT to extract data for 11 of 24 items (Figure 3).

Figure 3. Data extraction questions used in ChatGPT

1. What were the review's objectives, research questions and aims?
2. What type of review is this?
3. How many databases were searched in? (e.g., databases such as PubMED, MEDLINE)
4. What date was the database search?
5. What is the publication date range of all studies included in the review's synthesis?
6. How many studies were included in the synthesis of the review?
7. What countries were the studies included in the synthesis of the review conducted in?
8. What instrument was used to assess the studies included in the review's quality and/or bias?
9. What were the type of studies included in the synthesis of the review?
10. How many quantitative and qualitative studies were included in the review?
11. What methods were used to synthesise the review's findings?
